# croFGD: *Catharanthus roseus* Functional Genomics Database

**DOI:** 10.3389/fgene.2019.00238

**Published:** 2019-03-22

**Authors:** Jiajie She, Hengyu Yan, Jiaotong Yang, Wenying Xu, Zhen Su

**Affiliations:** State Key Laboratory of Plant Physiology and Biochemistry, College of Biological Sciences, China Agricultural University, Beijing, China

**Keywords:** *Catharanthus roseus*, co-expression network, functional module, gene function, monoterpene indole alkaloid

## Abstract

*Catharanthus roseus* is a medicinal plant, which can produce monoterpene indole alkaloid (MIA) metabolites with biological activity and is rich in vinblastine and vincristine. With release of the scaffolded genome sequence of *C. roseus*, it is necessary to annotate gene functions on the whole-genome level. Recently, 53 RNA-seq datasets are available in public with different tissues (flower, root, leaf, seedling, and shoot) and different treatments (MeJA, PnWB infection and yeast elicitor). We used in-house data process pipeline with the combination of PCC and MR algorithms to construct a co-expression network exploring multi-dimensional gene expression (global, tissue preferential, and treat response) through multi-layered approaches. In the meanwhile, we added miRNA-target pairs, predicted PPI pairs into the network and provided several tools such as gene set enrichment analysis, functional module enrichment analysis, and motif analysis for functional prediction of the co-expression genes. Finally, we have constructed an online croFGD database (http://bioinformatics.cau.edu.cn/croFGD/). We hope croFGD can help the communities to study the *C. roseus* functional genomics and make novel discoveries about key genes involved in some important biological processes.

## Introduction

*Catharanthus roseus*, a model plant of the Apocynaceae family, is best known for production of the bis-indole monoterpene indole alkaloids (MIAs). There are four important MIAs, vinblastine and vincristine used in the clinic as anti-cancer agents (Aslam et al., 2010), catharanthine which can reduce blood sugar content (Pan et al., 2012), and vindoline. MIAs belong to a class of terpenoid indole alkaloids (TIAs). Some TIAs exhibit strong pharmacological activities, whose production has beneficial effects on human health (Almagro et al., 2015). The biosynthesis of TIAs is regulated by several key transcription factors (TFs), such as ORCA3, ORCA2, WRKY, MYC, ZCT1, and BIS, which can enhance alkaloid production (Van Der Fits and Memelink, 2000; Suttipanta et al., 2011; Zhang et al., 2011; Li et al., 2013; Van Moerkercke et al., 2015; Rizvi et al., 2016). In addition to these key TFs, some hormones and transporters are essential for the regulation of TIA biosynthesis in *C. roseus* (Liu et al., 2017). Some external signals such as elicitor and jasmonate (JA) can regulate the activities of several TFs involved in TIA biosynthesis (Memelink and Gantet, 2007). Although much progress has been made in the field of TIAs, functions of some key genes and enzymes associated with the regulation of TIA biosynthesis are still unknown, which makes it difficult to understand the whole process. Notably, the release of the scaffolded genome sequence of *C. roseus* (Kellner et al., 2015), makes it possible to refine functional annotations of genes by integrating multidimensional data and existing methods.

The integration of biological information through gene expression profiling analysis can benefit to elucidating gene function (Noordewier and Warren, 2001). Transcriptomic datasets can be used to establish the gene expression profiles, which can provide some useful information for inferring gene regulatory relationship (Newton and Wernisch, 2014). Transcriptome analysis reveals that some genes involved in TIA biosynthesis are differentially expressed in leaf and root tissues, which can help understand specialized metabolic pathways in *C. roseus* (Verma et al., 2014). Integrated transcriptome and metabolome analysis can establish connections between genes and specialized metabolites, which can identify many genes involved in TIA synthesis and elucidate particular biological pathways (Rischer et al., 2006). Basing on transcriptomic datasets, the network construction can provide important biological knowledge, especially for digging out possible gene functions (Rhee and Mutwil, 2014).

Currently, there has been a plenty of transcriptomic datasets available on the public platform, which lay the foundation for the research in *C. roseus*. By considering all collected transcriptomic samples available together, co-expression network is applied to predicting gene functions on a large scale (Ma et al., 2014). Co-expression network analysis can mimic some important regulatory mechanism *in vivo* and thus discover key regulatory genes or functional modules. van Dam et al. (2017) excavated disease-related functional modules and annotated core genes based on co-expression network analysis. Considering that genes within a specialized metabolite pathway may form tight associations with each other in co-expression network, the method for connecting genes to specialized metabolic pathways in plant is effective, which can identify novel genes associated with specialized metabolic pathways (Wisecaver et al., 2017). Co-expression network analysis identified two missing enzymes, PAS and DPAS, necessary for vinblastine biosynthesis in *C. roseus*, which is important for understanding many other bioactive alkaloids (Caputi et al., 2018).

A growing number of studies have supported the utility of co-expression network analysis for inferring and annotating gene function, and excavating core genes involved in specific biological process. PlaNet used Heuristic Cluster Chiseling Algorithm (HCCA) to construct whole-genome co-expression networks for *Arabidopsis* and six important plant crop species (Mutwil et al., 2011). AraNet presented co-functional gene network for *Arabidopsis* and generated functional predictions for 27 non-model plant species using an orthologous-based projection (Lee et al., 2015). ATTED-II provided 16 co-expression platforms for nine plant species through combining the Pearson correlation coefficient (PCC) and mutual rank (MR) algorithm (Aoki et al., 2016). Our lab have published several functional genomics databases with co-expression network for plant species (Yu et al., 2014; You et al., 2015, 2016; Zhang et al., 2015; Tian et al., 2016; Ma et al., 2018). Besides, ccNET provided comparative gene functional analyses at a multi-dimensional network and epigenome level across diploid and polyploid *Gossypium* species based on the co-expression network (You et al., 2017). With the combination of transcriptomic and epigenomic data, MCENet provided global and conditional networks to help identify maize functional genes or modules associated with agronomic traits (Tian et al., 2018).

Here, we constructed a functional genomics database for *C. roseus* (croFGD). It provided three types of co-expression network, which allowed user to perform network search and analysis from a multi-dimensional perspective. Functional annotation information and several analysis tools were provided for functional prediction of the co-expression genes. Basing on co-expression network, we identified some functional modules which could be applied to the discovery of vital genes associated with agronomic traits. The integration of co-expression network analysis and functional module identification can be used to improve *C. roseus* gene function annotation and helpful for the functional genomics research. Besides, it can promote the research for the synthesis, metabolism of active substances and drug development.

## Materials and Methods

### Transcriptomic Data Source

There were 53 samples in *Catharanthus roseus* collected from the NCBI Sequence Read Archive (SRA), which covered different tissues (root, hairy root, shoot, stem, leaf, flower, seedling, and callus) and different treatments, such as methyl jasmonate (MeJA), peanut witches’ broom (PnWB) infection and yeast elicitor (Supplementary Table S1).

### Data Processing and Gene Expression Profiling Analysis

The *C. roseus* genome had a size of ∼500 Mb, and 33,829 protein-coding genes. All transcriptomic datasets were subjected to quality control using FastQC software (v0.10.1) (Brown et al., 2017). Those datasets with mapping rate <50% were filtered out. The sequence reads were mapped to the *C. roseus* reference genome (ASM94934v1) (Kellner et al., 2015) using Tophat (v2.0.10) software (Trapnell et al., 2009) with default parameters. Cufflinks (v2.2.1) (Trapnell et al., 2010) was used to calculate the FPKM (fragments per kilobase of transcript per million mapped reads) values with default parameters. And differentially expressed genes was calculated by Cuffdiff (v2.2.1) (Trapnell et al., 2013).

### Co-expression Network Construction

Pearson correlation coefficient is used to calculate correlation coefficient between two genes. MR represents high credible co-expression gene pairs after ranking the PCC. PCC is calculated based on the formula below. The more similar the expression pattern in samples between genes is, the higher the PCC score might be. MR is an algorithm basing on PCC, which takes a geometric average of the PCC rank from gene A to gene B and from gene B to gene A.

$$\begin{array}{c} PCC=\frac{\sum_{i=1}^{n}\left(x_{i}-\bar{x}\right)(y_{i}-\bar{y})}{\sqrt{\sum_{i=1}^{n}{\left(x_{i}-\bar{x}\right)}^{2}\cdot \sum_{i=1}^{n}{\left(y_{i}-\bar{y}\right)}^{2}}}\\ MR(AB)=\sqrt{\left(Rank(A\to B)\times Rank(B\to A)\right)} \end{array}$$

*X* or *Y* represents the FPKM value, and *n* represents the number of samples. MR ensures those co-expression gene pairs with low credibility will be filtered out, so the PCC and MR are combined to construct co-expression network. Here, all samples were used for the construction of global co-expression network. Among all samples, 44 samples without treatment were used to construct tissue-preferential network, and 32 samples with treatment and corresponding control were used to construct the treat-response network.

### Functional Module Identification and Parameter Selection

The Clique Percolation Method (CPM) (Adamcsek et al., 2006) was used to identify modules with nodes densely connected to each other in three types of co-expression networks, including global network, tissue-preferential network and treat-response network. Parameter selection was based on module number, module overlap rate and gene coverage rate. Here, we selected the *k* = 5 clique size for global co-expression network, which meant each module had at least five nodes and each node had co-expression relationship with each other (Supplementary Figure S2). In fact, one functional module could be regarded as a small network. Similarly, we selected the *k* = 6 clique size for tissue-preferential network and treat-response network. The functions of the modules were annotated through gene set enrichment analysis (GSEA) (Yi et al., 2013), including GO terms, gene families, plantCyc and KEGG pathways.

### The Identification of Orthologous Genes in *Arabidopsis*

Bidirectional blast alignments were conducted for the analysis of protein sequences between *C. roseus* and *Arabidopsis*. Our criteria for the identification of orthologous gene pairs were as follows: the top three hits in each bidirectional blast alignment were selected as the best orthologous pairs; in addition, orthologous pairs with an e-value less than 1E-25 were regarded as the second level.

### The Classification of Gene Family

Five main gene families, including TFs and regulator factors (TRs), carbohydrate-active enzymes, kinase, ubiquitin and cytochrome P450, were classified to improve limited functional annotation. TF/TRs and kinase family were identified mainly by iTAK tool (Zheng et al., 2016) based on the rule in PlnTFDB (Pérez-Rodríguez et al., 2009) and PlantsP Kinase Classification (Tchieu et al., 2003), respectively. The carbohydrate-active enzymes (CAZy) family (Lombard et al., 2014) was predicted through the method of orthologous search based on *Arabidopsis thaliana*. The enzymes were classified into six groups: glycoside hydrolases (GH), glycosyltransferase (GT), polysaccharide lyases (PL), carbohydrate esterase (CE), auxiliary activities (AA) and carbohydrate-binding modules (CBM). Ubiquitin family was identified through Hidden Markov Model (HMM) search based on models from UUCD (Gao et al., 2013). And cytochrome P450 family was predicted by orthologous relationship with *Arabidopsis* and the candidates were confirmed with ID of PF00067 by Pfam (Finn et al., 2014) search.

### Z-Score for Motif Analysis

Motif (*cis*-element) analysis tool is developed to identify significant motifs in one sequence or in the promoter region of interested gene list and thus predict possible functions. Z-score is a statistical measurement of the distance in standard deviations of a sample, which can act as a normalization method to eliminate the difference caused by background for different samples. So far, it is widely applied to calculating the *cis*-element significance (Endo et al., 2014).

The Z-score is calculated as:

$$\begin{array}{c} Z=\frac{\bar{X}-\mu }{\sigma /\sqrt{n}} \end{array}$$

$\bar{X}$ represents sum value of a motif in the promoter of one gene list. μ represents mean value of the same motif in 1,000 random gene lists with same scale. σ represents standard deviation of the 1,000 mean value based on random selection.

### Plant Materials and Growth Conditions

*C. roseus* seeds were planted in small pots and kept moistened until the seeds had germinated, and allowed to grow until they had three to five leaves, then transferred to a greenhouse (16 h light/8 h darkness, 28/25°C). For MeJA treatment, 100 μM MeJA was sprayed evenly on leaves and stem of well-growth plants. In order to prevent MeJA decomposition, leaves and stem with treatment and corresponding control were under darkness. After treatment for 6 and 24 h, the leaves and stem were harvested, immediately frozen in liquid nitrogen, and then stored at -80°C for use. Control samples were also harvested. Three biologically repeated samples were harvested.

### RNA Isolation and Quantitative Real Time RT-PCR

About 100 mg of tissue was ground in liquid nitrogen before isolation of the RNA. Total RNA was isolated using TRIZOL^®^ reagent (Invitrogen, Carlsbad, CA, United States) and purified using Qiagen RNeasy columns (Qiagen, Hilden, Germany). Reverse transcription was performed using Moloney murine leukemia virus (M-MLV; Invitrogen). We heated 10 μL samples containing 2 μg of total RNA, and 20 pmol of random hexamers (Invitrogen) at 70°C for 2 min to denature the RNA and then chilled the samples on ice for 2 min. We added reaction buffer and M-MLV to a total volume of 20 μL containing 500 μM dNTPs, 50 mM Tris-HCl (PH 8.3), 75 mM KCl, 3 mM MgCl_2_, 5 mM dithiothreitol, 200 units of M-MLV and 20 pmol random hexamers. The samples were then heated at 42°C for 1.5 h. The cDNA samples were diluted to 2 ng/μL for real time RT-PCR analysis.

For quantitative real-time RT-PCR, triplicate quantitative assays were performed on 1 μL of each cDNA dilution using the SYBR Green Master Mix with an ABI 7900 sequence detection system according to the manufacture’s protocol (Applied Biosystems). The gene-specific primers were designed using PRIMER3^1^. The amplification of 18S rRNA was used as an internal control to normalize all data (forward primer, 5′-CGGCTACCACATCCAAGGAA-3′; reverse primer, 5′-TGTCACTACCT CCCCGTGTCA-3′). Gene-specific primers were listed in Supplementary Table S2. The relative quantification method (ΔΔCT) was used to evaluate quantitative variation between replicates examined.

## Construction and Content

### Database Construction

**FIGURE 1:**
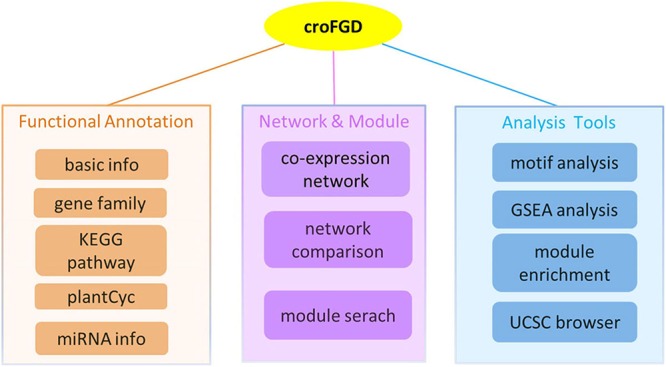
Database architecture. The croFGD database is divided into three main pieces: network & module, functional annotation, and analysis tools. The line with different colors indicates different pieces.

The database was constructed under the LAMP (Linux + Apache + Mysql + PHP) environment. It mainly contains three parts: (I) functional annotation, which includes gene family, KEGG pathway and miRNA detailed information, etc.; (II) network and module, including co-expression network search and analysis, network comparison and module search; (III) some analysis tools, mainly including *cis*-element enrichment analysis, GSEA, functional module enrichment analysis and UCSC Genome Browser visualization (Figure 1).

### Functional Annotation

We obtained the functional annotation information in *C. roseus* from the Dryad Digital Repository (Kellner et al., 2015). Among 33,829 protein-coding genes, 14,527 genes were annotated with 4,734 GO terms by blast2GO (Conesa and Gotz, 2008). 5,571 enzymes involved in 213 metabolism pathways were annotated by GhostKOALA (Kanehisa et al., 2016) from KEGG database. We mapped *C. roseus* protein sequences against CathaCyc (Van Moerkercke et al., 2013) using the BLASTP program and 2,421 enzymes involved in 513 metabolism pathways were annotated. Then we predicted 36,882 orthologous pairs between *C. roseus* and *Arabidopsis* through bidirectional blast alignment. There were a total of 1,035 plant motifs collected from the Plant *Cis*-acting Regulatory DNA Elements (PLACE) database (Higo et al., 1999), PlantCARE database (Rombauts et al., 1999), AthaMap database (Steffens, 2004) and literatures. Furthermore, we adopted the inparanoid algorithm (Sonnhammer and Östlund, 2015) and predicted 9,377 protein–protein interaction (PPI) pairs in *C. roseus* from over 18,000 experimentally validated PPI pairs in *Arabidopsis* integrated from several databases, such as BIOGRID (Chatr-Aryamontri et al., 2017), IntAct (Orchard et al., 2014) and related literature (Lumba et al., 2014). We also collected 227 miRNA sequence information derived from a literature (Shen et al., 2017), and then mapped these miRNA sequences against the whole-genome sequence using the GMAP program (Wu and Watanabe, 2005). Furthermore, 143 miRNA targets were identified by psRNATarget (Dai and Zhao, 2011). The miRNA detailed information mainly included location, sequence and structure, miRNA target and expression profiles in seedling after MeJA treatment (Supplementary Figure S3). Furthermore, we conducted the gene family classification and finally predicted 88 TFs/TRs families with 1,702 genes, 21 ubiquitin families with 1,192 genes, 98 cytochrome P450 families with 191 genes, 85 kinase families with 778 genes and 96 CAZy families with 1,505 genes (Table 1).

**Table 1:** Data collection and statistics in croFGD.

Database content	Number	Source	Reference
GO terms (genes)	55,505 (14,527)	Blast2GO tool	Conesa and Gotz, 2008
KEGG pathway (genes)	213 (5,571)	GhostKOALA tool	Kanehisa et al., 2016
PlantCyc (genes)	513 (2,421)	Blastp prediction	–
*Cis*-elements (motifs)	1,035	Database and literature collection	–
Orthologous pairs in *Arabidopsis* (genes)	36,882 (14,719)	Blast alignment	–
Transcription factor and regulators (members)	88 (1,702)	iTAK prediction	Zheng et al., 2016
Kinases (members)	85 (778)		
Carbohydrate-active enzymes (members)	96 (1,505)	Blast alignment	Lombard et al., 2014
Ubiquitin (members)	21 (1,192)	Blast alignment	Zhou et al., 2018
Cytochrome P450 (members)	98 (191)	the cytochrome p450 homepage	Nelson, 2009
Co-expression network nodes (%)	30,096 (88.9%)	PCC and MR	Aoki et al., 2016
Tissue-preferential network nodes (%)	29,808 (88.1%)		
Treat-response network nodes (%)	30,541 (90.3%)		
Protein–protein interaction pairs	9,377	InParanoid algorithm	Sonnhammer and Östlund, 2015
miRNA target modules	143	psRNAtarget prediction	Dai and Zhao, 2011
Function modules from global network (nodes)	2,310 (10,757)	CFinder tool	Adamcsek et al., 2006
Function modules from tissue-preferential network (nodes)	1,849 (12,090)		
Function modules from treat-response network (nodes)	2,177 (12,073)		

### Co-expression Network and Functional Module

A well-developed strategy with the integration of PCC and MR algorithm was widely applied to the construction of co-expression network (You et al., 2016, 2017; Obayashi et al., 2018; Tian et al., 2018). We used the 240 BP terms of GO associated with >4 and <20 genes to evaluate the networks. To get optimal gene pairs and evaluate the credibility of co-expression network, we selected different PCC thresholds of PCC > 0.7, PCC > 0.8, PCC > 0.9 and different MR thresholds of MR top3 + MR ≤ 30, MR top3 + MR ≤ 50, MR top3 + MR ≤ 100 to predict gene functions basing on selected GO terms and generated receiver operating characteristic (ROC) curves (Supplementary Figure S1). The larger the area under the curve (AUC) value of co-expression network is, the higher the credibility of the network will be. Finally, we selected the thresholds of PCC > 0.7 and MR top3 + MR ≤ 30 to filter out those co-expression gene pairs with low credibility to construct co-expression network. In total, there were 30,096, 29,808 and 30,541 nodes in global network, tissue-preferential network and treat-response network with gene expression view, which covered 88.9%, 88.1%, and 90.3% of genes in *C. roseus*, respectively (Table 1). All networks were visualized by Cytoscape 2.8 (Smoot et al., 2011).

Then we overlaid the gene expression value onto the co-expression network to identify whether genes in the network were expressed or not based on the minimum threshold FPKM value. To determine the minimum threshold of the gene expression value (FPKM) among all *C. roseus* samples (detailed mapping results are shown in Supplementary Table S3), the lowest 5% of all gene FPKM values in each sample and the standard deviation (SD) of each experimental group were computed. The mathematical formula “threshold = average (5% value) + 3 ^∗^ SD” (You et al., 2016, 2017) was used to calculate the minimum expression value of each experimental group. The minimum threshold of FPKM was 0.094. We identified differential expressed genes between treatment and control samples by the cutoff: |log_2_FC|≥ 1 and *p*-value ≤ 0.05. Tissue-preferential analysis in different tissues (root, hairy root, shoot, stem, leaf, flower, seedling, and callus) and treat-response analysis under three types of treatments (MeJA, PnWB infection and yeast elicitor) among five tissues (root, shoot, flower, callus, and hairy root) were supplied for the co-expression network analysis. Meanwhile, predicted miRNA target and PPI pairs were integrated into the network, and further analysis was provided for all members in the network, such as gene expression profiling analysis, GSEA, and *cis*-element analysis.

Furthermore, co-expression network could be used to perform modularized analysis and excavation for the discovery of agronomic trait-related vital gene and functional module. The CPM proposed to detect the overlapping communities in the complex network (Palla et al., 2005; Li et al., 2014), provided certain practicability for the discovery of key gene and module. Finally, we applied the algorithm and predicted 2,310, 1,849, and 2,177 functional modules in global network, tissue-preferential network and treat-response network in *C. roseus*, respectively (Table 1). The functions of these modules were annotated through GSEA (Yi et al., 2013). The entries which were not significant were filtered out by Fisher’s tests and multiple test correction method (FDR ≤ 0.05). These functional modules covered diverse functions such as vindoline and vinblastine biosynthesis, jasmonic acid biosynthesis, pathogen resistance and hormone response, etc.

### Analysis Tools

#### Gene Set Enrichment Analysis

Gene set enrichment analysis (Yi et al., 2013) is a powerful method for the functional annotation of interested gene list by computing the overlaps with well-defined background gene sets. Some categories of gene sets, such as GO terms, gene families, plantCyc and KEGG pathways, miRNA targets and functional modules identified from three types of network, were used as background gene sets. The significantly enriched gene set with FDRs ≤ 0.05 would be displayed on the GSEA result page.

#### Functional Module Enrichment Analysis

The tool was used to identify some functional modules from interested gene list especially in the network. The previously annotated miRNA target modules and functional modules identified from three types of network were used as background functional modules. The modules with FDRs ≤ 0.05 would be regarded as significantly enriched and the enrichment analysis result page included module annotation, module source, overlap gene number, and FDR value.

#### *Cis*-Element Enrichment Analysis

*Cis*-element (motif), a short conserved sequence, can be recognized by some TFs to regulate the expression levels of downstream genes. The tool was developed to identify motifs in a set of gene promoters and thus predict the function of gene set. The *cis*-element significance test is an algorithm using statistical method based on Z-score and *p*-value filtering (Yu et al., 2014) that can identify significant *cis*-regulatory elements in the promoter region of one gene. The promoter region was set as 3 kb in *C. roseus*. When scanned in the 3 kb promoter region of *C. roseus* genes, motifs with *p*-value ≤ 0.05 were significantly enriched on account of the frequency of motif occurrence.

#### Other Tools Supported in croFGD

A quick search, UCSC Genome Browser (Speir et al., 2016) visualization and a manual were provided for users. The search page mainly included gene detail search, gene function search, functional module search and orthologous search. The orthologous search allowed user to input one gene list in *Arabidopsis* to search for corresponding *C. roseus* genes.

## Function Application

### Comprehensive Exploration for the Function of 16OMT Gene

**FIGURE 2:**
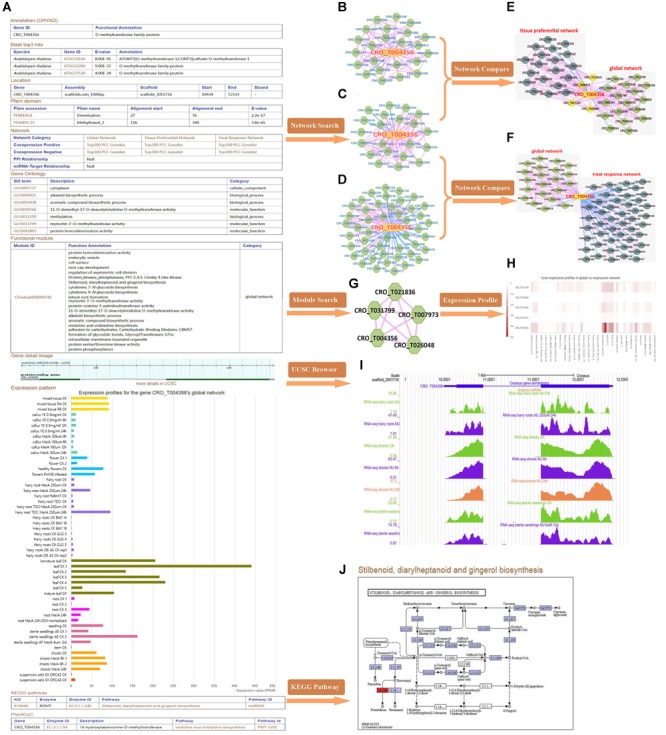
Comprehensive explorations for the function of *16OMT* (*CRO_T004356*) gene. **(A)** The detailed information of *16OMT* gene in *C. roseus*. Three types of co-expression network, including tissue-preferential network **(B)**, global network **(C)** and treat-response network **(D)**. In these networks, the node with yellow color represents the gene submitted initially, and the nodes with green color represent co-expressed genes; the edge with pink color links two genes with positive co-expression relationship; the edge with blue color links two genes with negative co-expression relationship. **(E)** Network comparison between global network and tissue-preferential network. The nodes with yellow color represent overlap genes between two networks, and the nodes with green and dark green color stand for unique genes in two networks, respectively. **(F)** Network comparison between global network and treat-response network. **(G)** The “CFinderADM000741” module. **(H)** Expression heatmaps of genes in “CFinderADM000741” module. **(I)** UCSC genome browser visualization. **(J)** Stilbenoid, diarylheptanoid, and gingerol biosynthesis pathway.

CRO_T004356 (*16OMT*), o-methyltransferase family member, which was reported to be involved in the biosynthesis of TIAs (Pandey et al., 2016; Yamamoto et al., 2016). Taking *16OMT* gene as an example, we explored possible function of the gene through the database. By gene detail search, we found that the gene: (I) was annotated with alkaloid biosynthetic process (GO: 0009821) and myricetin 3′-*O*-methyltransferase activity (GO: 0033799), etc.; (II) had two pfam domains: “Dimerisation (PF08100)” and “Methyltransf_2 (PF00891)” domains; (III) was mainly involved in vindoline and vinblastine biosynthesis; (IV) was relatively high in expression in leaf tissue (Figure 2A). We conducted network analysis for three types of co-expression network of *16OMT* gene including tissue-preferential network (Figure 2B), global network (Figure 2C) and treat-response network (Figure 2D). GSEA results for global network genes indicated that these genes might be involved in phenylpropanoid biosynthesis, vindoline and vinblastine biosynthesis. Network comparison results suggested that it was relatively conservative between global network and tissue-preferential network (Figure 2E), and there were great differences between global network and treat-response network (Figure 2F). Through module search, the gene in the module (Figure 2G) might be involved in vindoline and vinblastine biosynthesis, alkaloid biosynthetic process, and protein phosphorylation, *etc*. Therefore, *16OMT* gene might have diverse function in several biological processes like *hos1* gene (MacGregor and Penfield, 2015). The expression heatmaps of all genes in the module were included (Figure 2H). UCSC genome browser visualization (Figure 2I) indicated that most RNA-seq peaks were enriched in the genic region. Furthermore, stilbenoid, diarylheptanoid, and gingerol biosynthesis pathway was shown (Figure 2J).

### Co-expression Network Analysis for CPR Gene

**FIGURE 3:**
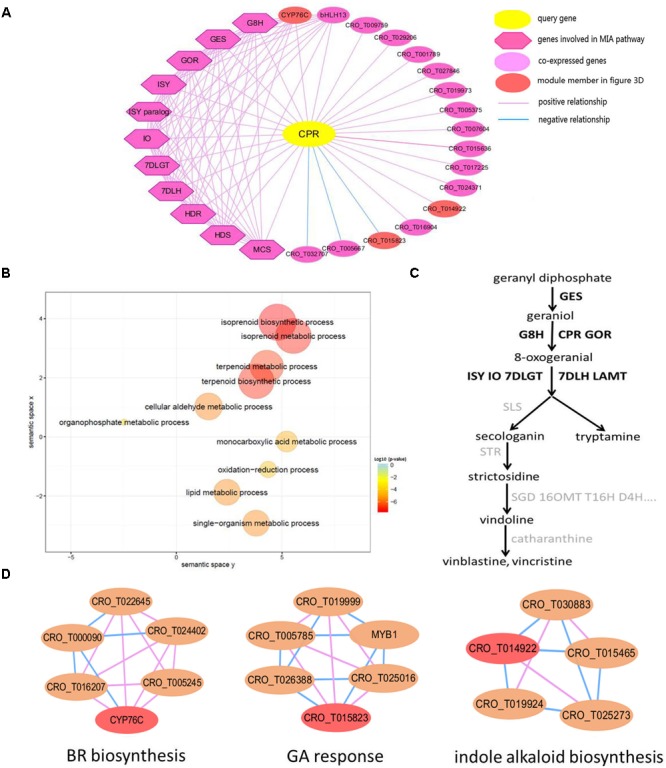
The global network of *CPR* (*CRO_T031702*) gene involved in MIA pathway. **(A)** Global network of *CPR* gene. The query gene *CPR* is highlighted by yellow, the blue line represents negative co-expression relationship between two genes, while the pink line represents positive co-expression relationship. The dark purple diamond represents several genes involved in the MIA biosynthesis pathway, such as *GES, 7DLH, GOR, HDS, G8H, ISY, MCS, HDR, 7DLGT*, and *IO*, which are co-expressed with *CPR* gene in the network, and the light purple circular represents other genes co-expressed with query gene. **(B)** Scatter plot of GO enrichment analysis results for all genes in *CPR* network. **(C)** The simplified MIA pathway. The bold represents the gene in *CPR* co-expression network. **(D)** Several functional modules related to genes in *CPR* network. The red node represents genes in *CPR* network.

CPR, NADPH–cytochrome P450 reductase, which is essential for the activation of cytochrome P450 enzymes, is critical for the biosynthesis of MIAs (Parage et al., 2016). The detailed information of all genes in the global network of *CPR* gene (Figure 3A) was listed in Supplementary Table S4. In the *CPR* network, some genes (*GES, 7DLH, GOR, HDS, G8H, ISY, MCS, HDR, 7DLGT* and *IO*) were involved in MIA biosynthesis pathway (Chebbi et al., 2014; Kumar et al., 2015). These genes were labeled with bold in the MIA biosynthesis pathway (Figure 3C). Through GO enrichment analysis (Tian et al., 2017) for all genes in the *CPR* network, the significantly enriched GO terms were associated with terpene biosynthetic process, and isoprenoid biosynthetic process (Figure 3B), which were related to MIA biosynthesis (Geu-Flores et al., 2012; Dugé de Bernonville et al., 2015). Through module enrichment analysis for all genes in *CPR* network, three genes (*CYP76C, CRO_T015823*, and *CRO_T014922*) in significantly enriched functional modules might be involved in brassinosteroid (BR) biosynthesis, gibberellic acid (GA) response and indole alkaloid biosynthesis, respectively (Figure 3D). Therefore, in addition to MIA biosynthesis, *CYP76C* and *CRO_T015823* also played important role in plant growth and development. Besides, *CRO_T014922* might also be involved in MIA biosynthesis together with other genes (*CRO_T019924, CRO_T030883, CRO_T015465*, and *CRO_T025273*) in the module (Figure 3D). Thus, in addition to the function of network, co-expressed genes might be involved in some other functions. Furthermore, co-expression analysis can be combined with module enrichment analysis to predict gene function effectively.

### Network Comparison Between Global Network and Tissue-Preferential Network of JAZ1 Gene

**FIGURE 4:**
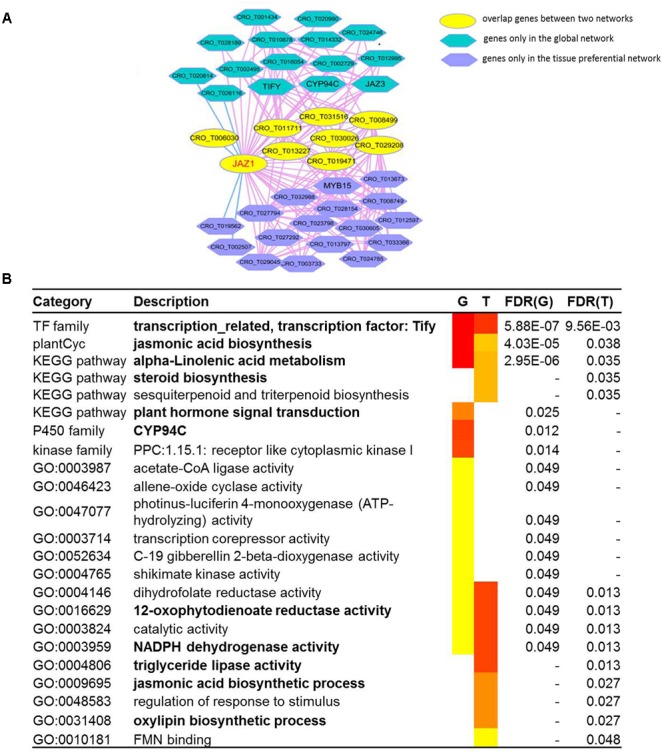
The network comparison between the global network and tissue-preferential network. **(A)** The comparison between the global network and tissue-preferential network of *JAZ1* (*CRO_T006982*). In the network comparison, the nodes with yellow color represents overlap genes between two networks, and the nodes with dark blue color represents genes only in tissue specific network, while the nodes with sky blue color represents genes only in global network. **(B)** GSEA results for two networks of *JAZ1*. The “G” represents global network, and the “T” represents tissue-preferential network.

JAZ1, a jasmonate-zim-domain protein, was discovered as repressors of jasmonate signaling, which was involved in TIA biosynthesis (Pan et al., 2018). We conducted network comparison between global network and tissue-preferential network of *JAZ1* (Figure 4A). The information of co-expressed genes in global network and tissue-preferential network was shown in Supplementary Table S5. We found that the two networks displayed different network structure. There were nine overlapped genes including *JAZ1* gene between two networks. Fifteen unique genes (including *TIFY, CYP94C*, and *JAZ3*) appeared in global network, while sixteen unique genes including *MYB15* appeared in tissue-preferential network. GSEA results for the genes in global network of *JAZ1* indicated that some gene sets were significantly enriched, such as jasmonic acid biosynthesis, alpha-linolenic acid metabolism, steroid biosynthesis and plant hormone signal transduction (Menke et al., 1999; Koo et al., 2014; Patra et al., 2018). GSEA results for the genes in tissue-preferential network of *JAZ1* illustrated that some gene sets were significantly enriched, such as jasmonic acid biosynthetic process, 12-oxophytodienoate reductase activity, NADPH dehydrogenase activity, triglyceride lipase activity and oxylipin biosynthetic process (Figure 4B) (Tani et al., 2008; Wallström et al., 2014; Wang et al., 2018). Based on the structure and function of the two networks of *JAZ1* gene, there were some conservation and differences between two networks. In *Arabidopsis*, cytochrome p450 family member *CYP94C1* and *CYP94B3* played important role in the regulation of jasmonate response (Niu et al., 2011; Heitz et al., 2012; Koo et al., 2014). In *Gossypium hirsutum, GhJAZ2* regulated the jasmonic acid signaling pathway by interacting with the R2R3-MYB transcription factor GhMYB25 (Hu et al., 2016). It needed further study whether the two genes *CYP94C* and *MYB15* coexpressed with *JAZ1* in two networks had similar function in *C. roseus* as in *Arabidopsis* and *Gossypium hirsutum*, respectively. These results indicated that network comparison is an effective approach to analyze gene function from the perspective of different networks.

### Treat-Response Network With Expression View After MeJA Treatment

**FIGURE 5:**
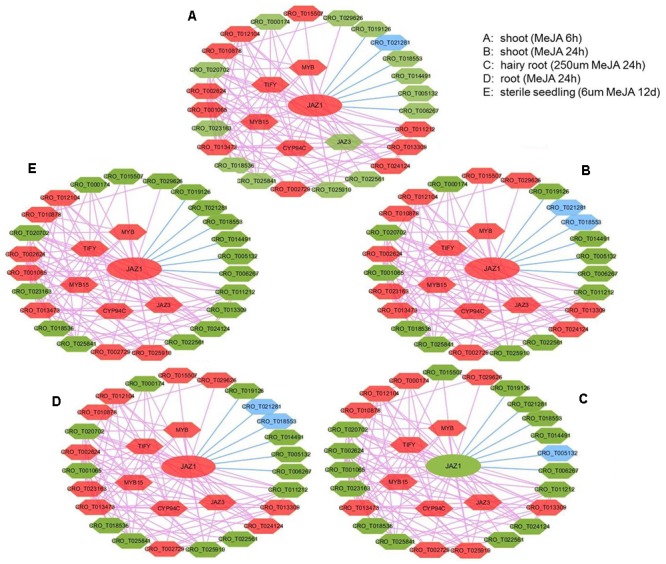
The expression view of *JAZ1* network after MeJA treatment in different tissues (shoot, root, hairy root, and seedling). The *JAZ1* network after MeJA treatment 6 h in shoot **(A)**, MeJA treatment 24 h in shoot **(B)**, 250 μM MeJA treatment 24 h in hairy root **(C)**, MeJA treatment 24 h in root **(D)**, 6 μm MeJA treatment 12 days in sterile seedling **(E)**. The red hexagon represents up-regulated genes, the blue hexagon represents down-regulated genes, and the green hexagon represents genes with no significant change in expression.

In *JAZ1* network with expression view after MeJA treatment in different tissues (shoot, root, hairy root, and seedling) (Figure 5), most genes had significant change in expression, such as *JAZ1, JAZ3, CYP94C, MYB, MYB15*, and *TIFY*. Detailed information for up and down-regulated genes in these networks was shown (Supplementary Table S6). In *C. roseus*, JAZ proteins could repress MYC2 and BIS1 to respond to JA signaling and then modulate MIA biosynthesis (Patra et al., 2018). In rice, enhanced expression of cytochrome p450 family member CYP94C2b could alleviate the jasmonate response and enhanced salt tolerance (Kurotani et al., 2015). In *Arabidopsis*, AtMYB44 could repress JA-mediated defense by activating the expression of *WRKY70* at transcriptional level (Shim et al., 2013). *PvTIFY10C* and *GsTIFY10* gene acted as a repressor in the JA signaling pathway in *Phaseolus vulgaris* and *Glycine soja* (Zhu et al., 2011; Aparicio-Fabre et al., 2013), respectively. We conferred that *CYP94C, MYB, MYB15*, and *TIFY* co-expressed with *JAZ1* might act as JA-response candidate genes in *C. roseus*. Furthermore, *CRO_T012104* (anthranilate synthase beta subunit), *CRO_T013473* (protein of unknown function), *CRO_T010878* (alpha/beta-hydrolases superfamily protein), *CRO_T002729* (allene oxide cyclase), and *CRO_T002624* (tryptophan biosynthesis) almost up-regulated under those five conditions, might also act as JA-response candidate genes. Taking treat-response network of *JAZ1* gene as an example, we selected six genes (*JAZ1, TIFY, MYB, CRO_T012104, CRO_T024124*, and *CRO_T002729*) for the real time RT-PCR validation (Supplementary Figure S4). These genes were up-regulated after MeJA treatment in shoot tissues and might act as JA-response genes. The qRT-PCR results indicated that these genes acted as JA-response genes in shoot tissues. This not only validated the accuracy of the predicted results, but also demonstrated the reliability of the network. Thus, treat-response network with expression view can clear display the dynamic change of gene expression in a network. Therefore, the co-expression network with multi-dimensional analysis can benefit to analyzing regulatory mechanisms in *C. roseus* development and stress response.

## Discussion

Our croFGD database aims to provide an online database server for the annotation and prediction of gene function. We constructed global network, tissue-preferential network and treat-response network with expression view, which covered almost 90% of gene in *C. roseus* and identified more than 6,000 functional modules. The annotation of these functional modules covered vindoline and vinblastine biosynthesis, jasmonic acid biosynthesis, hormone response and pathogen resistance, etc. The network analysis strategy, functional module annotation and integrated method could improve and refine gene function annotation from diverse perspectives to some extent. For some crops, it could be applied to excavate important functional module related to agronomic traits, which would be beneficial for genetic breeding.

Through some analysis tools supported in croFGD, we can excavate key genes involved in some important biological processes and predict gene function. In comprehensive exploration for the function of *16OMT* (Figure 2), we found that the gene might have complex function, like *hos1* gene (MacGregor and Penfield, 2015). In global network of *CPR*, some genes were involved in MIA biosynthesis, such as *GES, 7DLH, GOR, G8H, ISY*, and *7DLGT* (Figure 3A). The integration of co-expression network analysis and module enrichment analysis can be benefit to predicting gene function effectively and refining gene annotation. Basing on network comparison between two networks of *JAZ1*, there were certain similarities and differences whether in the structure or in the function of two networks (Figure 4). In addition, function of two genes *CYP94C* and *MYB15* needed further research. In treat-response network of *JAZ1* gene with expression view after MeJA treatment in different tissues, we identified several possible JA-response candidate genes (Figure 5 and Supplementary Table S6), which was experimentally validated by real time RT-PCR (Supplementary Figure S4). These results would be beneficial to understanding some molecular regulatory mechanisms in *C. roseus*, such as MIA biosynthesis and jasmonic acid biosynthesis, *etc*.

Comparative co-expression network analysis between species is an effective approach to predict gene function and improve functional annotation (Pathania et al., 2016). We conducted network comparison for gene list with PCC ranks in the top 300 between *C. roseus* and *Arabidopsis* (obtained from ATTED-II) (Supplementary Figure S5). High similarity between co-expression network of *JAZ1* in *C. roseus* and *AT1G19180* (*JAZ1*) in *Arabidopsis* not only demonstrated the reliability of co-expression network, but also illustrated the conservation of *JAZ1* gene function between these two species.

Based on co-expression network with multi-dimensional level, predicting functional module and refining gene function is an effective strategy, which can be used to identify more key genes and regulatory modules when we focus on a detailed biological process. Interestingly, co-expression network is highly associated with the regulation of epigenetic modification, such as DNA methylation (El-Sharkawy et al., 2015) and H3K4me3 (Farris et al., 2015), which can be integrated to understand detailed molecular mechanism, such as the biosynthesis of specific metabolites. There is a certain correlation between co-expression network and metabolic network, the integration of which can be used to predict key enzyme-coding genes and metabolites (Chen et al., 2013), and contribute to better understanding of the molecular mechanisms related to plant metabolic pathway (Rischer et al., 2006; Coneva et al., 2014).

Notably, there are additional limitations and possible improvements for croFGD database. Firstly, the release of the chromosome-level genome of *C. roseus* in the future, will greatly promote the research on functional genomics. Secondly, more RNA-seq samples of other tissues and treatments could be integrated into the co-expression network construction on the transcriptomic level, which will be beneficial to excavate gene function and improve the whole genome annotation in *C. roseus*. Thirdly, epigenomic data, such as ChIP-seq and DNase-seq data, can be integrated to improve the annotation of *cis*-elements and predict gene function. Furthermore, more accurate data, such as gene families, new type of non-coding RNAs, KEGG pathway and GO terms, needs to be integrated, too. Our croFGD database will be updated regularly, and we hope the database can help the community study the functional genomics and yield novel insights into the molecular regulatory mechanisms.

## Data Availability

Publicly available datasets were analyzed in this study. This data can be found here: https://www.ncbi.nlm.nih.gov/sra.

## Author Contributions

JS performed gene functional annotation, functional module identification, and database construction. HY performed data collection and the co-expression network construction. JY gave advice a lot about the web server. WX gave advice for the application of the co-expression network and some key functional module identification. ZS and WX supervised the project. All authors read and approved the final manuscript.

## Conflict of Interest Statement

The authors declare that the research was conducted in the absence of any commercial or financial relationships that could be construed as a potential conflict of interest.

## Footnotes

1 http://frodo.wi.mit.edu/primer3/input.htm
