# Supplementary material for: croFGD: Catharanthus roseus Functional Genomics Database
Source: Front Genet. 2019 Mar 22;10:238. doi: 10.3389/fgene.2019.00238 (PMC6438902; doi:10.3389/fgene.2019.00238)
Supplement: Supplementary file 1 [file Data_Sheet_1.doc]

Supplementary Material

croFGD: *Catharanthus roseus* Functional Genomics Database

Jiajie She^†^, Hengyu Yan^†^, Jiaotong Yang, Wenying Xu*, Zhen Su*

^†^These authors contributed equally to this work.

*** Correspondence:** Zhen Su: [zhensu@cau.edu.cn](mailto:zhensu@cau.edu.cn); Wenying Xu: [x_wenying@yahoo.com](mailto:x_wenying@yahoo.com)

# Supplementary Figures and Tables

## Supplementary Figures


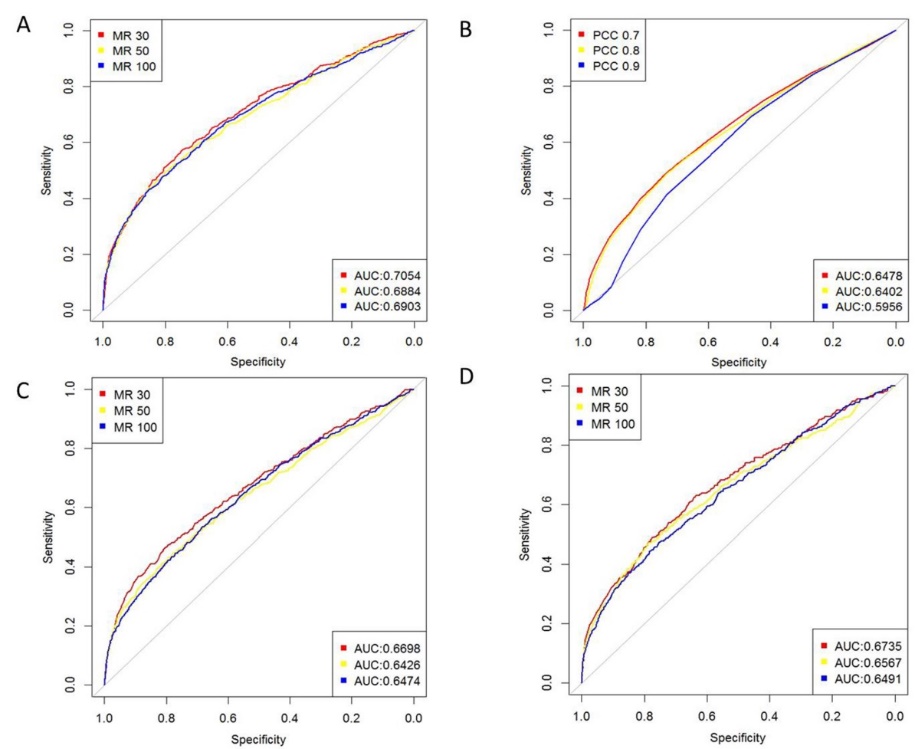


**Supplementary Figure 1** ROC curves of co-expression networks with different PCC thresh-olds and with different MR thresh-olds.

(**A**) ROC curves of global co-expression network with different MR thresholds (MR top3 + MR ≤ 30, MR top3 + MR ≤ 50, MR top3 + MR ≤ 100). (**B**) ROC curves of global co-expression network with different PCC thresholds (PCC > 0.7, PCC > 0.8, PCC > 0.9). (**C**) ROC curves of tissue-preferential network with different MR thresholds. (**D**) ROC curves of treat-response network with different MR thresholds.

**
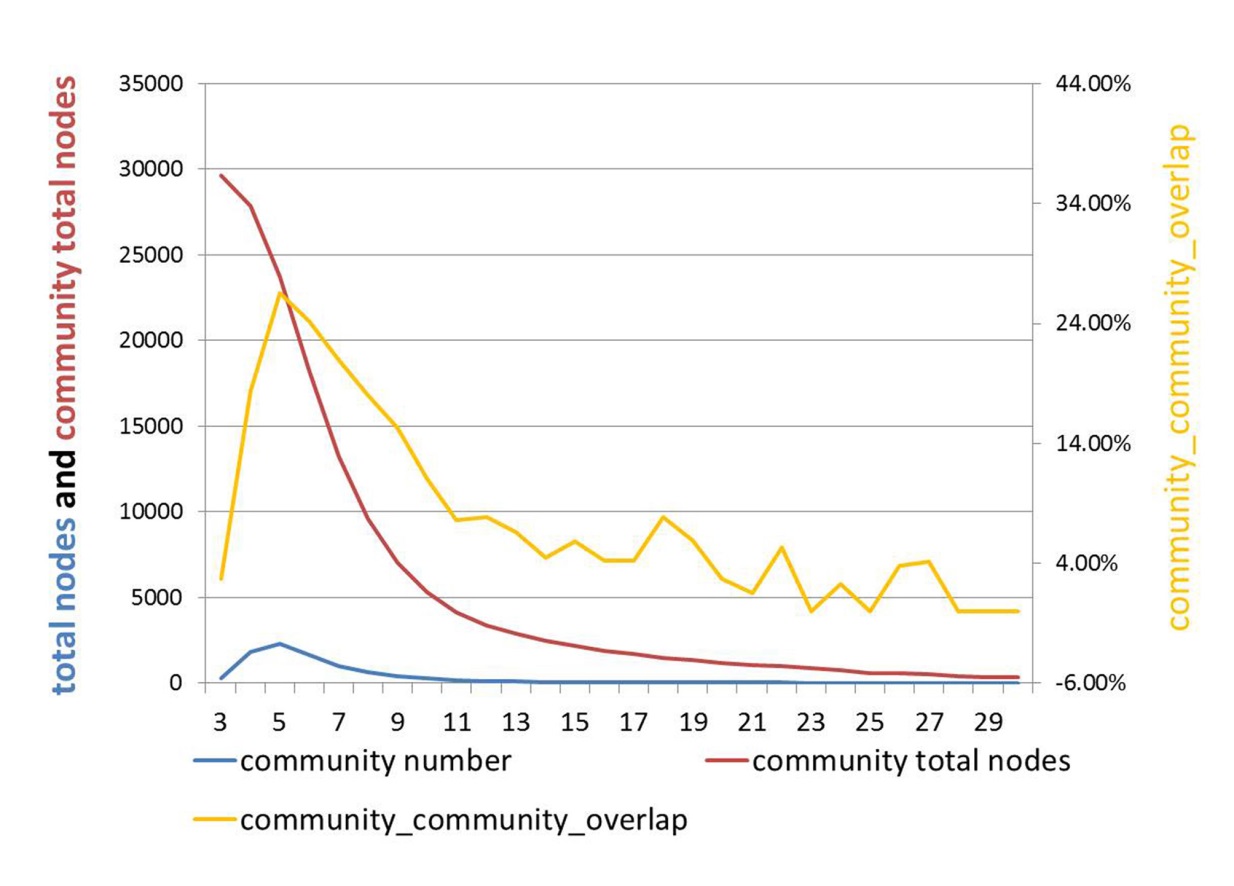
**

**Supplementary Figure 2** Proper selection for the size of functional module

The CPM algorithm was applied to calculate communities of different k-clique sizes (from k = 3 to k = 30). Statistics of the communities of different k-clique sizes, including community number, community-community overlap and community total nodes are compared. Here, community number represents the number of community of a selected k-clique size; community-community overlap represents the number of nodes contained by two overlapping communities; community total nodes represents the total genes contained in a given k-clique size. The left y-axis represents the community number and community total nodes, while the right y-axis represents the community-community overlap ratio (number of nodes contained by two overlapping communities/community total node).


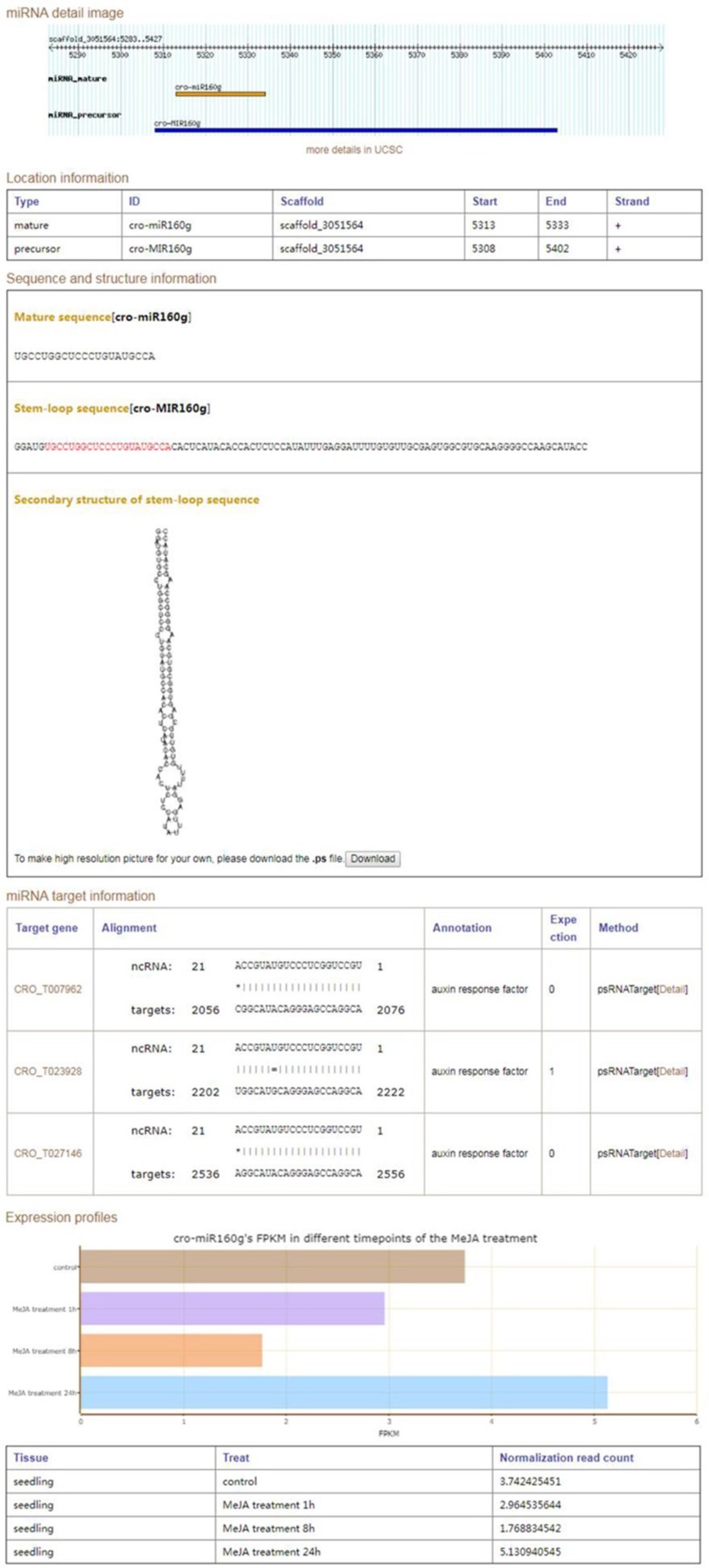


**Supplementary Figure 3** The detailed information of miRNA *cro-miR160g*

The detailed information of miRNA *cro-miR160g* mainly includes miRNA detail image, location information, sequence and structure information, miRNA target information and expression profiles.


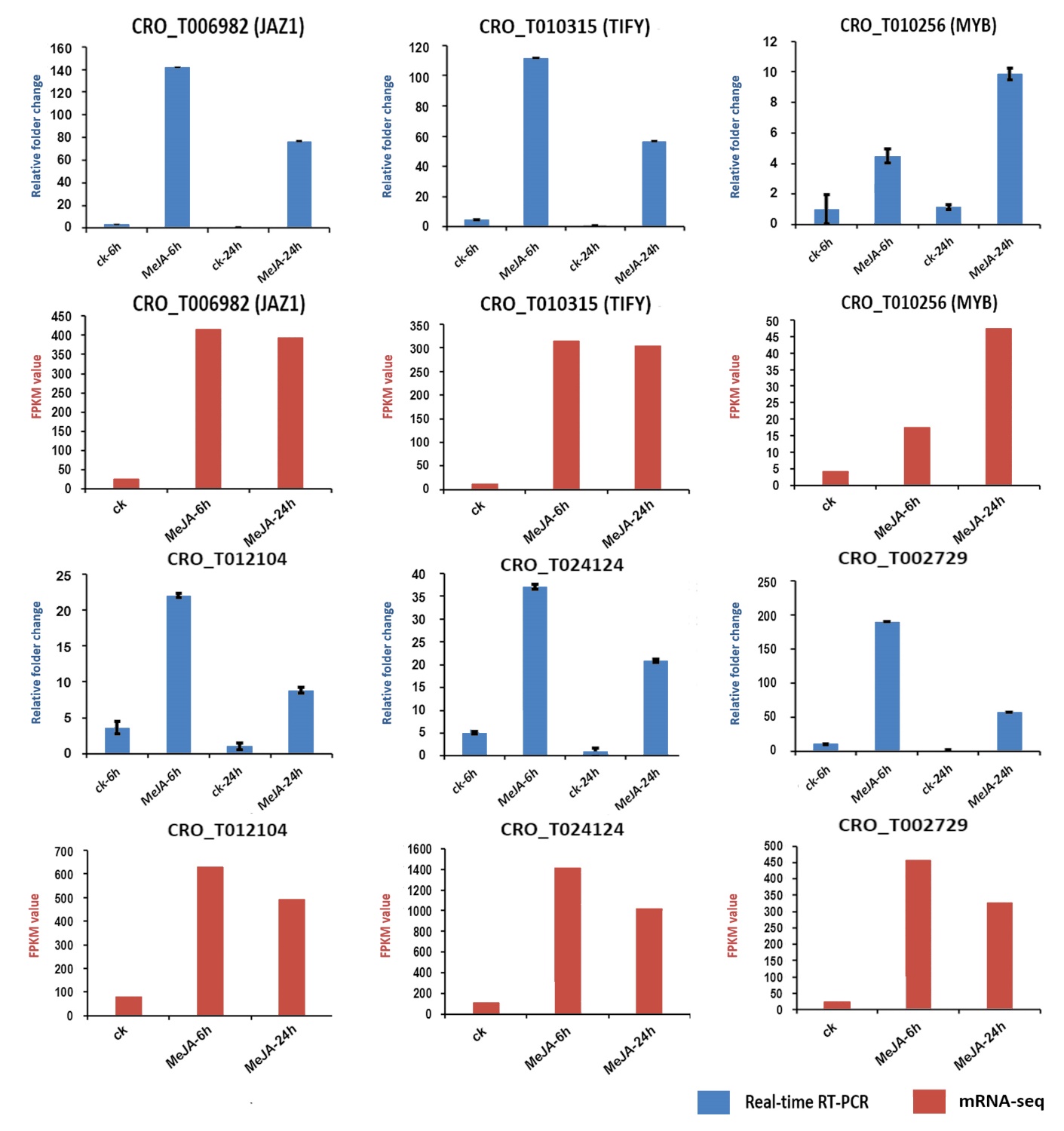


**Supplementary Figure 4** Quantitative real-time RT-PCR validation for selected genes.

Six genes were selected for quantitative real-time RT-PCR to validate the expression patterns after 6h and 24h MeJA treatment in shoot. The blue bars represent the relative intensity of real-time RT-PCR from three biological replicates, and the red bars represent the expression level (FPKM) of six genes. The quantitative real-time RT-PCR primers for each gene are listed in **Table S2**.


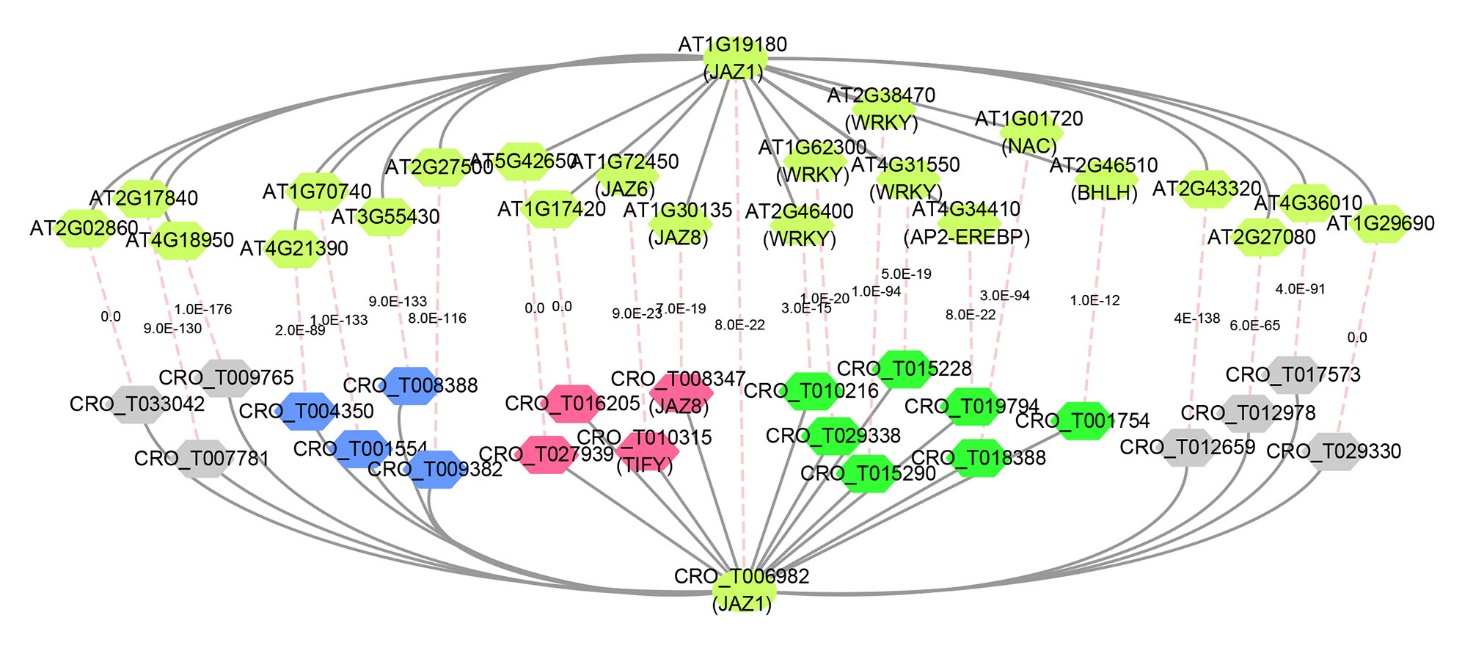


**Supplementary Figure 5** Comparison of global co-expression networks between *CRO_T006982* (*JAZ1*) in *C. roseus* and *AT1G19180* (*JAZ1*) in *Arabidopsis*.

Dotted lines link orthologous gene pairs between *C. roseus* and *Arabidopsis*, and the number in the middle of the dotted lines represents the e-value of the BLAST alignment result for orthologous gene pairs between *C. roseus* and *Arabidopsis*.

## Supplementary Tables (in separate excel file)

Table S1. Summary of RNA-seq datasets collected in *C. roseus*.

Table S2. Primer list of genes for qRT-PCR.

Table S3. The mapping results of RNA-seq datasets in *C. roseus*.

Table S4. The detailed information of all genes in *CPR* network.

Table S5. The information of co-expressed genes in global network and tissue-preferential network.

Table S6. The information of up/down-regulated genes in treat-response networks (**Figure 5**).
